# Effect of Superabsorbent Polymer Size on Strength and Shrinkage in Concrete Mixtures

**DOI:** 10.3390/polym17141942

**Published:** 2025-07-16

**Authors:** Wissawin Arckarapunyathorn, Pochpagee Markpiban, Raktipong Sahamitmongkol

**Affiliations:** 1 Department of Civil Engineering, King Mongkut’s University of Technology Thonburi (KMUTT), Bangkok 10140, Thailand; 2 Department of Civil Engineering, College of Engineering, Rangsit University, Pathumthani 12000, Thailand

**Keywords:** superabsorbent polymer, concrete, shrinkage, compressive strength, elastic modulus

## Abstract

This study investigates the influence of superabsorbent polymer (SAP) particle size on the mechanical and shrinkage behavior of concrete. Five concrete mixtures were prepared using SAPs with varying size ranges: 150–300 µm, 300–600 µm, 600–1800 µm, and a blended mix combining 300–600 µm and 600–1180 µm. The primary focus was on evaluating compressive strength, elastic modulus, autogenous shrinkage, drying shrinkage, and total shrinkage. The mechanical performance and dimensional stability were measured at different curing ages, and microstructural analysis was conducted using X-ray fluorescence (XRF) at 7 days to examine changes in chemical composition. Results showed that smaller SAP sizes contributed to more homogeneous internal curing, improved hydration, and higher matrix density. In contrast, larger SAP particles were more effective in reducing shrinkage but slightly compromised strength and stiffness. This study emphasizes the importance of selecting appropriate SAP particle sizes to balance mechanical integrity and shrinkage control, contributing to the development of high-performance concrete with reduced cracking potential.

## 1. Introduction

Polymer materials have become increasingly important in the construction industry due to their diverse properties, such as versatility, durability [1], and lightweight nature. These materials are widely applied in cement additives, reinforcement materials, adhesives, insulation, and protective coatings [2]. Commonly used polymers include polyurethane (PU), polyisobutylene (PIB), and rubber, each valued for their resilience and adaptability to various environmental conditions [2]. For example, PU is often used as a sealant in window and door frames to enhance energy efficiency and moisture resistance [2]. Due to their low density, strong chemical resistance, and insulating characteristics, polymers can improve structural service life as well as performance in harsh environments [1,2]. The ongoing development of smart and self-healing polymers continues to expand their role in sustainable and cost-effective construction solutions [3,4]. Ongoing breakthroughs in smart and self-healing polymers continue to broaden and diversify their role in sustainable and affordable construction solutions.

The surge in demand for high-performance concrete (HPC) in infrastructure and structural applications has rekindled interest in the challenges associated with early-age shrinkage and cracking. Due to their low water-to-cement (w/c) ratios, HPC mixtures often tend to regularly undergo self-desiccation, which leads to autogenous shrinkage and consequently increases the risk of early cracking. This issue is particularly critical in elements with high surface-to-volume ratios or restrained geometries, such as slabs, bridge decks, and precast members. Traditional external curing methods typically fall short in addressing these challenges, especially in dense or complex structural elements where water ingress is limited. To address these limitations, internal curing has emerged as a promising strategy for mitigating early-age shrinkage. Among various internal curing agents, superabsorbent polymers (SAPs) have gained significant attention due to their ability to absorb large volumes of water during mixing and slowly release it during hydration. This delayed water release helps maintain internal relative humidity and supports continued hydration. This happens especially at later stages when external moisture may not penetrate effectively. Beyond shrinkage control, internal curing using SAPs can also enhance cement hydration, reduce porosity, and improve microstructural development—factors that contribute to overall concrete performance and durability. However, optimizing SAP use requires careful consideration of factors such as particle size, dosage, and interaction with other mix components.

Among polymer-based technologies, superabsorbent polymers (SAPs) have gained considerable traction in cement and concrete research. SAPs are cross-linked hydrophilic materials capable of absorbing and retaining large volumes of water, often several hundred times their own weight [5,6]. Their water-retention capacity, combined with their ability for sustained water release, makes them exceptionally effective in facilitating internal curing in concrete mixtures [7,8]. Internal curing guarantees sustained moisture availability during early-age hydration, notably when external curing methods are deficient or unrealistic [9]. This is crucial in low water-to-cement ratio mixtures, where rapid moisture loss can cause early-age cracking, shrinkage, and incomplete hydration [10]. In addition to improving shrinkage behavior, SAPs may also influence the formation of hydration products and the development of the pore structure, which in turn affects mechanical performance [11,12]. Consequently, SAPs are increasingly regarded not only as shrinkage-reducing agents but also as multifunctional modifiers that can enhance the overall performance and sustainability of advanced concrete systems [6,13].

Several studies published since 2015 have validated the role of SAPs as effective internal curing agents for minimizing autogenous and drying shrinkage in cement-based materials [2,3,4]. SAPs function by temporarily storing part of the mixing water and then gradually releasing it as hydration proceeds, especially after capillary pores empty out due to self-desiccation [6,14]. This process helps mitigate internal stress buildup and suppresses early-age microcrack formation, especially in high-performance or low w/c mixtures where traditional curing may be inadequate [10,15].

The effectiveness of SAPs correlates with their physical properties, particularly particle size and absorption capacity. Hasholt et al. [8] categorized SAPs according to their swelling kinetics, water retention capacity, and distribution within the cement matrix. Their findings revealed that smaller SAP particles tend to swell faster and distribute more uniformly, promoting early hydration, but offering a shorter duration of internal curing. Conversely, while larger SAP particles can retain water longer and extend curing effects, they leave behind large voids that compromise the integrity of the hardened matrix [11,12].

Research by Zhu et al. [16] and Dudziak et al. [17] further demonstrated that SAP-modified concrete exhibited fewer shrinkage cracks, which translated into improved long-term durability in real-world applications such as slabs, pavements, and tunnel linings. However, the mechanical response of SAP-modified mixtures has shown mixed outcomes. Several authors have reported that improper SAP selection, particularly excessive dosage, or the use of coarse particles—can reduce compressive strength and elastic modulus due to macrovoid formation [13,16]. In contrast, studies using optimized SAP sizes and dosages have found improved strength-to-shrinkage performance ratios, especially in ternary or blended cement systems [13,18].

Recently, research has shifted towards exploring hybrid or multi-scale internal curing systems, where SAPs are used in combination with shrinkage-reducing admixtures (SRAs), supplementary cementitious materials (SCMs), or lightweight aggregates [4,5,8,13]. These approaches aim to create synergy by combining the water-buffering effect of SAPs with other physical or chemical shrinkage control mechanisms. Lee et al. [13] noted that pairing SAPs with fly ash or silica fume enhanced hydration kinetics will improve pore structure refinement. However, the interaction between SAP particle size and other admixtures remains an area requiring further investigation.

Though substantial work has been conducted on SAPs, most studies almost exclusively address shrinkage mitigation independently. Few have systematically evaluated the trade-offs between shrinkage control and mechanical performance under consistent effective w/c ratios. Moreover, the effects of SAP particle size on hydration product formation, especially during early-age curing, have not been comprehensively linked to microstructural or chemical analysis techniques such as XRF. This research seeks to address these gaps by providing a comparative evaluation of concrete formulations modified with SAPs of varying particle sizes while maintaining consistent mix design parameters.

This study focuses on examining the effect of SAP particle size on key concrete properties, including compressive strength, shrinkage behavior, and hydration-related changes in chemical composition. Since autogenous shrinkage primarily occurs within the first 7 days of curing, the hydration response was analyzed at this age using X-ray fluorescence (XRF) to record early-age chemical changes. This approach facilitates the assessment of the effect of SAP sizes on cement hydration by examining variations in elemental composition, especially those associated with hydration products. Concrete mixtures with different SAP particle size ranges, including a blended configuration, were compared to determine an optimal integration strategy that minimizes shrinkage without significantly compromising mechanical performance. The conclusions drawn from this research are intended to bolster ongoing developments in polymer-modified concrete and provide practical guidance for the application of SAPs in structural and infrastructure projects where shrinkage control is critical.

## 2. Materials and Methods

### 2.1. Materials

The primary binder used in this study was ordinary Portland cement (OPC) conforming to ASTM C150 Type I [19]. The chemical compositions are shown in Table 1.

The physical properties of aggregates are shown in Table 2. River sand was used as a fine aggregate. The specific gravity, fineness modulus, and water absorption of the river sand were 2.63, 2.57, and 0.94%, respectively. The fine aggregate was sieved through a No. 4 (opening 4.75 mm) sieve to remove large particles. Crushed limestone was used as a coarse aggregate with a maximum size of 20 mm. The specific gravity, fineness modulus, and water absorption of the crushed limestone were 2.61, 6.49, and 0.75%, respectively. The fine and coarse aggregates, in the saturated surface dry (SSD) condition, were used according to the ASTM C128 standard [20]. Tap water was used as mixing water.

The SAPs employed in this study were potassium-based superabsorbent polymers with varying particle size ranges. A covalently cross-linked acrylamide/acrylic acid copolymer was used as the SAPs. The maximal absorption capacity is 106.71 g (water)/g (dry SAP) at 3 h. Four size categories were selected to explore the effects of both fine and coarse particles: 150–300 µm (CS300), 300–600 µm (CS600), 600–1180 µm (CS1180), and a blended mix (CS600+1180) containing the 300–600 µm SAP and the 600–1180 µm SAP by mass. The physical properties and SAPs particle size categories by mass are shown in Table 3 and Table 4, respectively.

### 2.2. Mix Proportions

The five concrete mix proportions used in this study included one control mix and four SAP-modified mixes with varying particle size configurations. The control mix, labeled C35, contained no SAP and was designed to achieve a target 28-day compressive strength of 35 MPa using a fixed water-to-cement (w/c) ratio of 0.40. The SAP-containing mixes were designated as CS300, CS600, CS1180, and CS600+1180, where the suffix indicates the SAP particle size range. Specifically, CS300 incorporated SAPs sized 150–300 µm; CS600 used SAPs sized 300–600 µm; CS1180 included 600–1800 µm SAPs; and CS600+1180 contained a blend of SAPs sized 300–600 µm and 600–1180 µm. The SAP dosage was fixed at 0.2% by mass of cement for all SAP-containing mixtures. To account for the water absorbed by the SAPs during mixing, a correction was applied to reduce the free mixing water, thereby ensuring a consistent effective w/c ratio of 0.40 across all mixes. This adjustment allowed the effects of SAP particle size on hydration, shrinkage, and mechanical performance to be isolated and evaluated under controlled conditions. The mix proportions are shown in Table 5.

### 2.3. Sample Preparation

The mixing and curing procedures were adopted from our previous study [21]. In brief, all dry materials, including cement, aggregates, and SAPs, were premixed before gradually adding the mixing water. To account for water absorption by SAPs, a correction was applied to maintain a consistent effective water-to-cement ratio of 0.40. After casting, specimens were demolded after 24 h and cured in lime-saturated water at 23 ± 2 °C until the designated testing age.

### 2.4. Testing Procedures

#### 2.4.1. Compressive Strength

Concrete cubes (150 mm × 150 mm × 150 mm) were prepared for each mixture. After demolding at 24 h, the specimens were divided into three groups and subjected to different curing regimes until the day of testing, in order to observe the effect of curing conditions on strength development:

- Water curing: Specimens were immersed in water.
- Sealed curing: Specimens were wrapped in polyethylene sheets.
- Air curing: Specimens were stored in laboratory air with a relative humidity of approximately 50–60%.

Compressive strength tests [5,11] were conducted at 7, 28, and 42 days in accordance with ASTM C39 [22]. Three specimens per curing method were tested for each age, and the average values were reported.

#### 2.4.2. Elastic Modulus

The static elastic modulus of concrete was determined in accordance with ASTM C469 [23] using cylindrical specimens (100 mm × 200 mm). The test was performed at 28 days on specimens under three curing conditions: water, sealed, and air curing. A compressometer was attached to each specimen to measure longitudinal strain under loading.

To determine the influence of SAP and curing conditions on stiffness, the experimental results for elastic modulus were compared with their theoretical equivalent values derived from the ACI 318-19 equation.

#### 2.4.3. Shrinkage Tests

Three types of shrinkage were evaluated in this study: autogenous, drying, and total shrinkage. The measurement procedure followed the method described in our previous study [21]. Shrinkage behavior was assessed using sealed prism specimens (75 mm × 75 mm × 285 mm) exposed to controlled environmental conditions (relative humidity ≈ 50%, temperature ≈ 23 °C) in accordance with ASTM C157 [24]. Total shrinkage was evaluated on unsealed specimens of the same dimensions, exposed to ambient laboratory conditions. Length changes were monitored over a 42-day period using a digital length comparator.

#### 2.4.4. X-Ray Fluorescence (XRF) Test

X-ray fluorescence (XRF) analysis was performed on powdered samples at 7 days to determine the oxide composition of the cement matrix and identify hydration [8,9]-related chemical changes between SAP-modified and control mixtures.

## 3. Results

### 3.1. Compressive Strength

**Figure 1:**
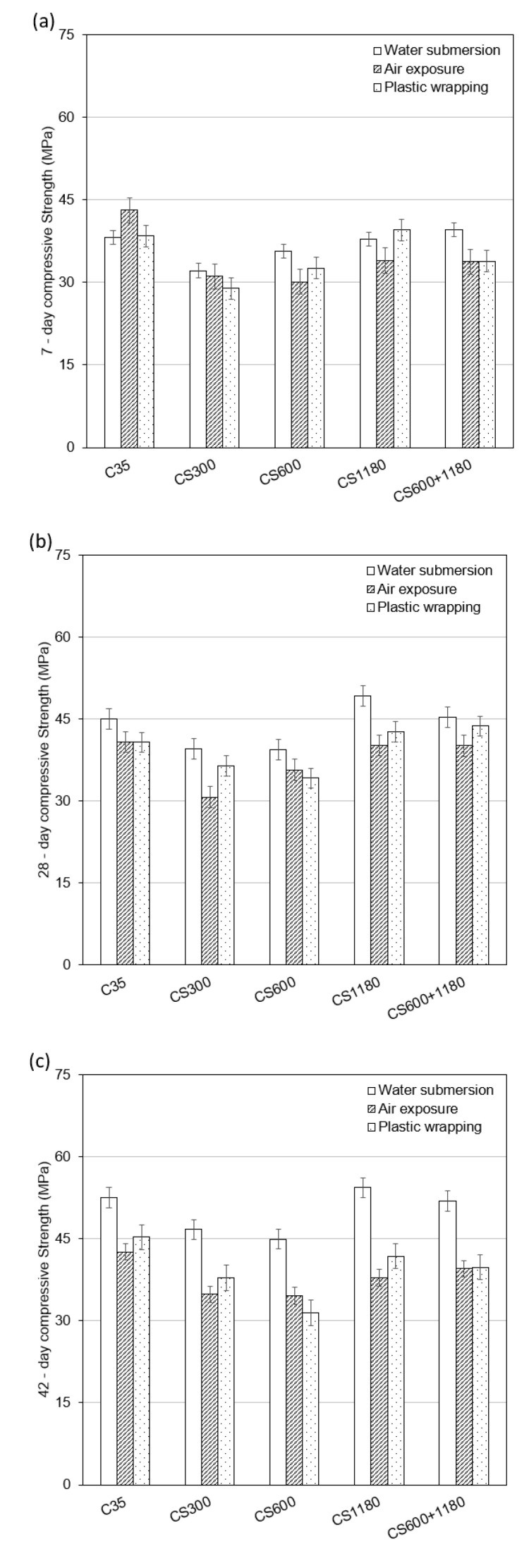
Compressive strength of concrete mixtures. (**a**) 7-day (**b**) 28-day and (**c**) 42-day.

The compressive strength test results for all five mixtures are shown in Figure 1. At 7 days (see Figure 1a), the control mix (C35) achieved a strength of 31.2 MPa. In comparison, CS300 and CS600 recorded strengths of 28.9 MPa and 27.5 MPa, respectively, while the coarser SAP mixes, CS1180 and CS600+1180, reached 25.8 MPa and 27.1 MPa. The trend sustained itself into later stages. By 28 days (see Figure 1b), the control attained 43.0 MPa, while the SAP-containing mixtures ranged from 36.2 MPa (CS1180) to 39.8 MPa (CS300). At 42 days (see Figure 1c), all mixtures showed further strength development, but the SAP-modified concretes consistently exhibited lower compressive strength than the control.

The reduction in strength observed earlier (at 7 days) in the process is due in part to the formation of macrovoids following the release of water from SAP particles. These voids act as discontinuities within the cement matrix, disrupting the transmission of mechanical stress. Coarser SAP particles like mix CS1180 tend to leave behind larger and more unevenly distributed voids, thereby magnifying the decline in strength.

In contrast, finer SAP particles, such as those used in CS300, offer two distinct advantages. First, they absorb and release water more quickly, which supports cement hydration during the early stages when internal curing is most beneficial. Second, their smaller size leads to more uniform dispersion and less disruptive void formation within the matrix. This results in a better strength retention profile relative to coarser SAPs. The superior performance of CS300, especially at 28 (see Figure 1b) and 42 days (See Figure 1c), highlights the potential of fine SAPs for balancing hydration support and microstructural integrity.

These findings are consistent with previous studies by Lee et al. [13], who reported improved strength retention when SAP sizes were maintained below 300 µm. They also align with general observations in the literature that while internal curing enhances long-term hydration, it may reduce early-age strength depending on SAP characteristics such as particle size, absorption rate, and swelling behavior.

### 3.2. Elastic Modulus of Concrete

**Figure 2:**
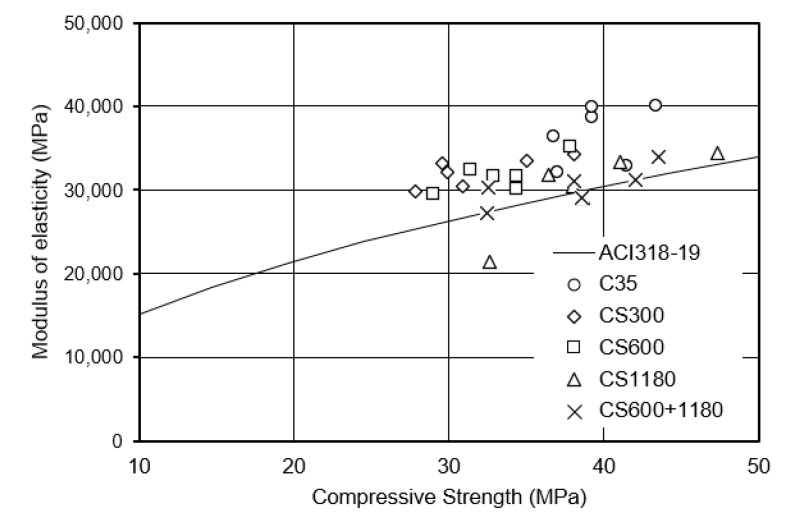
Elastic modulus of concrete mixtures.

The static modulus of elasticity values at 7 and 28 days are presented in Figure 2. The control concrete (C35) exhibited the highest stiffness, with a 28-day modulus of 28.2 GPa. In contrast, SAP-modified mixtures showed a gradual reduction in stiffness with increasing SAP particle size. CS300 (150–300 µm) and CS600 recorded the values 26.5 GPa and 25.2 GPa, respectively. The coarsest mix, CS1180 (600–1800 µm), had the lowest modulus at 23.4 GPa, while the blended mix (CS600+1180) yielded 25.0 GPa.

For comparison, the elastic modulus of the control concrete estimated using the ACI 318-19 equation was approximately 31.0 GPa based on its 28-day compressive strength. All measured values, including the control, were lower than the ACI prediction, with greater deviation observed in SAP-modified concretes. This can be attributed to increased internal porosity from SAP-induced voids, which the empirical ACI formula does not account for.

The reduction in stiffness is primarily due to pores formed as SAPs swell and release water, then shrink during hydration. Larger SAPs create larger, more localized voids, disrupting matrix continuity and reducing resistance to deformation. Conversely, finer SAPs (CS300) result in smaller, more evenly distributed pores, which less severely affect stiffness while still promoting internal curing.

These trends align with the findings of Huang et al. [6], who associated lower modulus values with increased porosity and microcracking near SAP particles. The blended mix showed a moderate reduction in stiffness, suggesting that combining SAP sizes can help balance internal curing efficiency and mechanical performance.

### 3.3. Shrinkage Behaviors

Concrete shrinkage was assessed based on total shrinkage, autogenous shrinkage, and drying shrinkage. Total shrinkage is defined as the overall dimensional change that stems from internal self-desiccation and moisture loss to the environment. To expand comprehension of the underlying mechanisms behind shrinkage reduction in SAP-modified concrete, autogenous and drying shrinkage were monitored independently under sealed and unsealed conditions, respectively.

#### 3.3.1. Total Shrinkage

**Figure 3:**
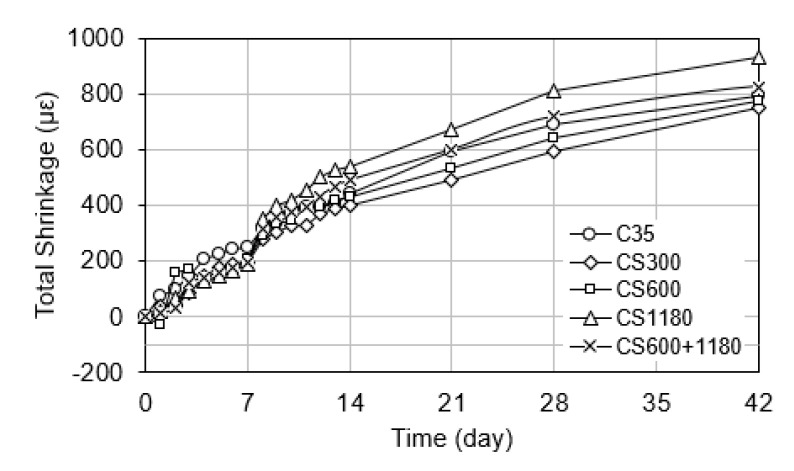
Total shrinkage of concrete mixtures.

Total shrinkage represents the combined effect of autogenous and drying shrinkage over the entire 42-day period. As shown in Figure 3, the control mix (C35) exhibited the highest total shrinkage, reflecting the lack of internal curing and full exposure to both self-desiccation and moisture loss. In contrast, all SAP-modified mixtures demonstrated notable reductions in total shrinkage, consistent with findings reported in previous studies [2,6,18,25].

Among the SAP mixtures, CS300, which contained the finest SAP particles (150–300 µm), showed the lowest total shrinkage, followed closely by the blended CS600 mix. CS600+1180 and CS1180 exhibited higher total shrinkage values, which were higher than the control. These results are in agreement with the work of Dudziak et al. [17], who demonstrated that SAPs effectively mitigate shrinkage by providing continuous moisture release during early and intermediate curing stages. Similarly, De Meyst et al. [25] confirmed that SAPs reduce total shrinkage and crack risk in high-performance concrete elements exposed to drying environments.

The trend observed in this study reinforces the established understanding that the internal curing efficiency of SAPs depends heavily on particle size and absorption kinetics. Coarser SAPs retain water longer, releasing it gradually as hydration and drying progress, thus maintaining internal humidity for an extended period [4,5]. This prolonged release supports both early-age and long-term hydration, reducing cumulative shrinkage. On the other hand, finer SAPs tend to release absorbed water quickly, offering hydration benefits primarily during early stages, with diminishing effects thereafter [10,14].

The blended mixture (CS600+1180) demonstrated a balanced shrinkage profile, combining early internal curing from smaller SAPs with prolonged moisture retention from larger ones. This result is supported by Mignon et al. [2], who highlighted that SAP blends with complementary absorption profiles can provide extended curing effects while minimizing porosity and strength loss. Overall, the total shrinkage performance of the SAP-modified concretes confirms that SAP selection based on particle size distribution can be a strategic approach for controlling volumetric deformation in drying-sensitive structural applications.

#### 3.3.2. Autogenous Shrinkage

Autogenous shrinkage principally derives from self-desiccation during cement hydration. It is more pronounced in low water-to-cement ratios, where internal water is inadequate for complete saturation/hydration. In sealed conditions, the consumption of capillary water leads to internal drying, resulting in volume contraction. This mechanism is particularly pronounced during the first 7 days, making early-age monitoring essential.

**Figure 4:**
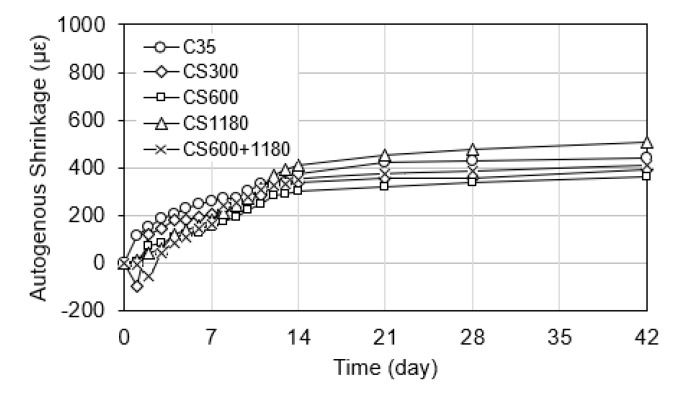
Autogenous shrinkage of concrete mixtures.

As shown in Figure 4, the control mixture (C35) and CS1180 experienced the highest level of autogenous shrinkage, with rapid deformation observed within the first few days. In contrast, all SAP-modified mixtures exhibited significant reductions in autogenous shrinkage. Among them, the CS600, CS300, and CS600+1180 mixtures showed the most effective mitigation, indicating the benefit of prolonged internal curing from SAPs.

These observations are consistent with the internal curing mechanism described by Jensen and Hansen [5], where SAPs serve as localized water reservoirs that gradually release moisture as hydration proceeds. The size and structure of SAPs directly affect the rate of water release and, therefore, the shrinkage control period. Larger SAPs deliver a slower and more prolonged water supply, maintaining internal humidity and decreasing early-age stress development [10,26].

The reduced autogenous shrinkage in SAP-containing mixes also aligns with findings from Li and Chen [26]; these researchers showed/established that under low w/c concrete conditions, SAPs increase internal relative humidity and suppress capillary tension. Similarly, Hasholt et al. [8] and Liu et al. [12] demonstrated that the internal curing capacity of SAPs leads to better hydration continuity and lower shrinkage strain under sealed conditions. While fine SAPs promote rapid hydration and early strength gain, they may not sustain internal humidity long enough to mitigate autogenous shrinkage over extended curing periods.

In summary, the results confirm that SAP particle size significantly influences the autogenous shrinkage behavior of concrete. Coarser particles enhance internal curing duration, effectively mitigating early-age volume change, while finer SAPs are more suitable for short-term hydration support. These findings support the use of particle-size-engineered SAPs for tailored shrinkage control in high-performance concrete.

#### 3.3.3. Drying Shrinkage

Drying shrinkage occurs when hardened concrete loses moisture to the surrounding environment, typically after the end of the initial curing phase. It is caused by water evaporation from capillary pores, leading to capillary tension and volumetric contraction. In this study, drying shrinkage was monitored starting at day 7, after the sealed specimens were exposed to ambient laboratory conditions.

**Figure 5:**
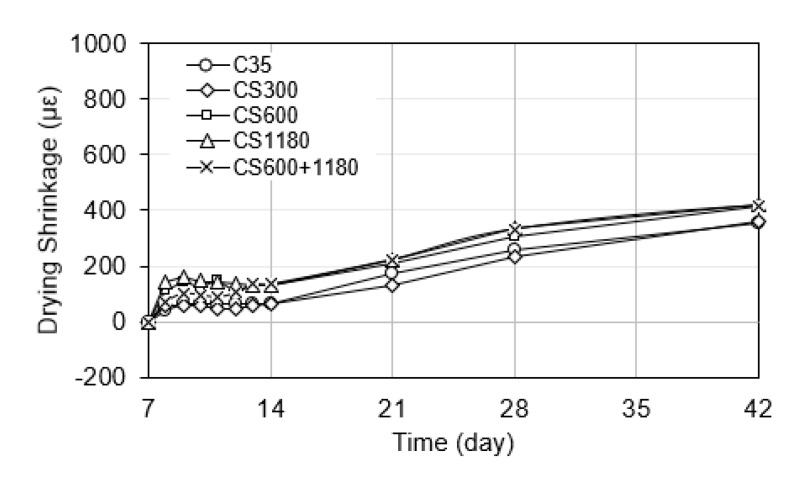
Drying shrinkage of concrete mixtures.

As illustrated in Figure 5, the CS600+1180 exhibited the highest drying shrinkage over the 42-day monitoring period. The CS300 mixtures showed the most substantial reductions in drying shrinkage. This is attributed to the more even dispersion rate of the finer SAP particles, which extended the internal curing effect beyond the initial hydration phase and into the drying period. Their sustained water retention delayed the development of internal moisture gradients, thereby reducing capillary stress and shrinkage.

In contrast, the CS600, CS1180, and CS600+1180 mix demonstrated the least reduction in drying shrinkage among the SAP-containing mixes. While these larger particles improved early-age hydration and reduced autogenous shrinkage, their rapid water release was largely completed during the sealed phase, offering limited benefit once the specimens were exposed to the drying environment. This behavior aligns with observations by Li et al. [27] and Kong et al. [10], who noted that the water retention duration of SAPs plays a more critical role in drying shrinkage control than absorption capacity alone.

Drying shrinkage mitigation is closely tied to the timing of internal water availability relative to moisture loss to the environment. SAPs that retain water beyond 7 days contribute more effectively to shrinkage control under drying conditions. This reinforces the importance of selecting SAPs not only based on absorption rate and size, but also on their water release profile and compatibility with anticipated curing and exposure conditions.

Overall, the results confirm that SAP particle size influences drying shrinkage behavior by regulating the duration and effectiveness of internal curing. The superior performance of the blended mix (CS600+1180) suggests that combining SAPs with different size ranges can provide both early-age hydration support and extended moisture retention, making it a promising approach for minimizing drying-induced deformation in concrete.

### 3.4. X-Ray Fluorescence (XRF) Results

X-ray fluorescence (XRF) analysis was conducted on paste samples extracted from each mixture at 7 days to evaluate the influence of SAP particle size on early-age hydration. The goal was to assess whether internal curing by SAPs altered the chemical composition of hydration products, as seen in Table 6. Special emphasis was placed on calcium oxide (CaO) and silicon dioxide (SiO_2_), key indicators for assessing cement hydration progress/stages/kinetics.

As shown in Table 6, the CS300 and CS600 mixtures exhibited slightly higher levels of CaO and SiO_2_ compared to the control and the mixes containing coarser SAPs. This suggests that finer SAP particles contributed to more complete early hydration, likely due to their faster water release kinetics. An important characteristic of fine SAPs is their capacity to rapidly furnish additional moisture to mixtures during the early stages of hydration. This phenomenon and phase promote the formation of calcium silicate hydrate (C–S–H), the primary adhesive agent in concrete. These results align with observations from Zhao et al. [14] and Chen et al. [9], who reported enhanced cement hydration and increased C–S–H development in SAP-modified systems with optimized water availability.

In contrast, the mixtures with coarser SAPs (CS1180 and CS600+1180) showed slightly lower CaO and SiO_2_ contents at 7 days. This may be attributed to the delayed water release from larger SAPs, which prolongs internal curing and supports hydration at later ages rather than early stages. While beneficial for long-term shrinkage control, this slower hydration progression is reflected in the reduced early-age reaction rates captured by XRF.

These chemical findings support the mechanical and shrinkage results discussed earlier. Mixtures with finer SAPs, such as CS300, demonstrated higher early compressive strength, which correlates with greater hydration product formation at 7 days. Meanwhile, mixtures with coarser SAPs exhibited more gradual strength development and improved shrinkage control, suggesting extended hydration beyond the XRF analysis-captured timeframe.

Although the magnitude of chemical variation observed by XRF was relatively small, the trends reinforce the conclusion that SAP particle size influences the rate and extent of cement hydration. These results are consistent with findings from Huang et al. [6] and Li and Chen [26], who noted that SAP-modified systems exhibit altered hydration dynamics depending on SAP size and dispersion characteristics. This study’s combined mechanical, shrinkage, and chemical data highlights the link between internal curing behavior and early-age cement chemistry. The findings emphasize the importance of matching SAP properties with performance objectives in concrete design.

## 4. Discussion

### 4.1. Relationship Between SAP Size and Shrinkage

The results clearly demonstrate a strong correlation between SAP particle size and shrinkage reduction. Finer SAP particles, such as those used in CS300, yielded the most significant reduction in both autogenous and drying shrinkage. This is attributed to the more even dispersion rate of the finer SAP particles, which extended the internal curing effect beyond the initial hydration phase and into the drying period, supporting more even dispersion of internal curing and mitigating self-desiccation. These findings align with previous studies by Jensen and Hansen [5] and Dudziak et al. [17], who reported extended internal humidity control and lower shrinkage with SAPs.

In contrast, coarser SAPs (e.g., CS1180) have a less uniform dispersion. As a result, they were less effective in controlling long-term shrinkage, particularly under drying conditions. This behavior reflects the balance between water release rate and internal curing duration that defines the shrinkage mitigation capacity of SAPs.

Interestingly, the blended SAP mixture (CS600+1180) demonstrated a more balanced shrinkage response than either of the single-size SAP mixtures. This synergistic behavior can be explained by the combination of rapid early-age curing from the finer SAPs (300–600 µm) and extended curing from the coarser fraction (600–1180 µm). The result supports the concept proposed by Mechtcherine et al. [4] and Mignon et al. [2], who emphasized the role of SAP gradation in designing shrinkage-resistant concrete mixtures.

### 4.2. Relationship Between Strength Performance and Shrinkage

A key challenge in SAP-modified concrete is the inherent trade-off between shrinkage mitigation and mechanical strength. SAPs improve internal curing but also introduce voids into the matrix after water release, which can compromise strength development. This effect was most evident in the CS1180 mixture, which, despite showing excellent shrinkage reduction, exhibited the lowest compressive strength. The larger voids left by coarse SAPs act as flaws that reduce load-bearing capacity/The larger voids created by coarse SAPs act as defects that degrade/undermine load-bearing capacity.

In contrast, the CS300 mixture, incorporating finer SAPs, demonstrated higher compressive strength across all ages. The smaller and more evenly distributed voids caused less disruption to the matrix, while the rapid water release supported early hydration and densification. This substantiates findings by Lee et al. [13], who observed that SAPs under 300 µm could retain strength performance without compromising internal curing benefits.

The CS600+1180 blend offered a moderate reduction in strength compared to the control but performed better than CS1180, indicating that blending SAP sizes can help balance mechanical and shrinkage performance. This design flexibility is valuable in practice. For non-structural elements such as slabs or precast panels, where dimensional stability is prioritized over strength, coarser SAPs may be preferred. Conversely, for load-bearing elements like beams and columns, finer or blended SAPs may provide a better compromise. Snoeck and De Belie [3] also advocated SAP selection based on structural performance requirements, supporting this tailored approach.

### 4.3. Relationship Between XRF Composition and Compressive Strength

XRF analysis provided chemical insight into the hydration behavior of the different mixtures, complementing mechanical and shrinkage data. By day 7, mixtures containing finer SAPs (CS300 and CS600) demonstrated marginally higher/greater CaO and SiO_2_ contents than the control and coarse SAP mixtures. These oxides are associated with hydration product formation, particularly calcium silicate hydrate (C–S–H), and their higher presence indicates more complete early-age hydration.

The faster water release from finer SAPs likely enhanced cement particle reaction rates during the critical early hydration period, which in turn contributed to higher early strength. This observation aligns with Huang et al. [6] findings, which correlated higher CaO content with improved strength in SAP-modified mortars. Conversely, the coarser SAP mixes (e.g., CS1180) exhibited lower CaO and SiO_2_ levels at 7 days, consistent with delayed hydration due to slower internal water release.

These trends validate the perspective that SAP particle size impacts hydration kinetics. Finer SAPs promote early hydration and strength development, while coarser SAPs prolong hydration and primarily benefit shrinkage control. Hasholt et al. [8] also reported that SAP-induced internal curing improved the microstructure at the interfacial transition zone (ITZ), contributing to improved bonding when hydration was well-supported. Thus, XRF analysis not only confirms mechanical test data but also provides valuable micro-level insight into the role of SAPs in early-age cement chemistry.

## 5. Conclusions

This study investigated the influence of SAP particle size on the mechanical and shrinkage behavior of concrete, validated by chemical analysis at early hydration stages. Based on the experimental results, the following conclusions can be drawn:

- Finer SAPs, such as those used in the CS300 mixture (150–300 µm), released absorbed water more rapidly, promoting early-age hydration. This resulted in higher compressive strength and elastic modulus compared to mixes with coarser SAPs.
- Finer SAPs, such as those used in the CS300 mixture (150–300 µm), yielded the most significant reduction in both autogenous and drying shrinkage.
- The mixture with blended SAP sizes, CS600+1180 (combining 300–600 µm and 600–1180 µm), exhibited the most balanced performance, considering significant shrinkage reduction while maintaining acceptable mechanical properties.
- XRF analysis at 7 days showed that mixtures with finer SAPs had slightly higher CaO and SiO_2_ content, indicating more complete hydration and supporting the observed mechanical performance trends.
- These results reinforce the importance of selecting appropriate SAP sizes—or combinations thereof—based on project-specific performance requirements, particularly when balancing shrinkage control with mechanical strength.

While the current study provides clear evidence of the relationship between SAP particle size and concrete performance in terms of shrinkage, hydration, and strength, further investigation is encouraged to expand on these findings. Future research may focus on microstructural analysis aimed at understanding the distribution and influence of SAP-induced porosity on performance. This includes evaluating how different SAP sizes affect internal pore connectivity and matrix densification. In addition, assessing durability-related properties such as chloride ion diffusion, water permeability, and carbonation resistance would help contextualize the long-term impact of SAP-modified pore structures in service environments. These findings would support broader application of SAP size optimization strategies in performance-based concrete design, particularly for structures exposed to harsh or moisture-sensitive conditions.

## Figures and Tables

**Table 1:** Chemical properties of cement.

Chemical Composition (%)	Portland Cement Type 1
SiO_2_	20.90
Al_2_O_3_	4.80
Fe_2_O_3_	3.40
CaO	65.40
K_2_O	0.45
TiO_2_	0.26
Others	4.79

**Table 2:** Physical properties of aggregates.

Properties	River Sand	Crushed Limestone
Specific Gravity (SSD)	2.63	2.61
Fineness Modulus	2.57	6.49
Water Absorption (%)	0.94	0.75

**Table 3:** Physical properties of SAPs.

Properties	SAPs
Specific Gravity (OD)	1.34
Water Absorption (g (water)/g (dry SAP))	106.71

**Table 4:** SAPs particle size categories by mass.

Sample	SAPs Particle Size Categories by Mass (%)		
	150–300 µm	300–600 µm	600–1180 µm
CS300	100	-	-
CS600	-	100	-
CS1180	-	-	100
CS600+1180	-	50	50

**Table 5:** Mix proportions.

Sample	Mix Proportions (kg/m^3^)					
	Cement	River Sand	Crushed Limestone	Water	SAP	Internal Curing Water
C35	525	710	860	210	-	-
CS300	525	710	860	210	0.35	38
CS600	525	710	860	210	0.35	38
CS1180	525	710	860	210	0.35	38
CS600+1180	525	710	860	210	0.35	38

**Table 6:** XRF results of concrete mixtures at 7 days.

	C35	CS300	CS600	CS1180	CS600+1180
MgO	2.6	3.5	1.9	2.6	3.0
Al_2_O_3_	1.0	0.9	1.0	0.8	0.9
SiO_2_	12.1	7.5	10.4	6.6	9.1
P_2_O_5_	0.7	0.5	0.8	0.7	0.7
K_2_O	1.9	1.8	1.7	1.6	1.7
CaO	75.9	78.0	75.6	79.2	73.4
TiO_2_	0.4	0.5	0.6	0.5	0.5
Fe_2_O_3_	5.5	5.5	6.6	6.4	8.9
SO_3_	0.0	1.9	1.4	1.6	1.9
Total (%)	100.0	100.0	100.0	100.0	100.0

## Data Availability

The original contributions presented in this study are included in the article. Further inquiries can be directed to the corresponding author.
